# Radiological education in the era of artificial intelligence: A review

**DOI:** 10.1097/MD.0000000000038552

**Published:** 2024-05-31

The article “Radiological education in the era of artificial intelligence: A review,”^[1]^ which published in Volume 102, Issue 1 of *Medicine*, is being retracted due to concerns that were raised with some overlap in content from “Challenges of radiology education in the era of artificial intelligence,”^[2]^ including the overall structure and reference list. All manuscripts are subjected to a plagiarism check prior to final acceptance using iThenticate software; however, it has become evident that the text was phrased differently enough to avoid detection in some key passages, such as the first paragraph of the introduction, which is structurally very similar to Gorospe-Sarasúa et al, including many of the same cited references. As a result, the Publisher no longer has confidence in the originality of content and ethical standards of this review.
